# Validity of an Inertial Measurement Unit System to Measure Lower Limb Kinematics at Point of Contact during Incremental High-Speed Running

**DOI:** 10.3390/s24175718

**Published:** 2024-09-02

**Authors:** Lisa Wolski, Mark Halaki, Claire E. Hiller, Evangelos Pappas, Alycia Fong Yan

**Affiliations:** 1 Faculty of Medicine and Health, The University of Sydney, Sydney, NSW 2050, Australiaalycia.fongyan@sydney.edu.au (A.F.Y.); 2 School of Health and Biomedical Sciences, Royal Melbourne Institute of Technology, Melbourne, VIC 3000, Australia

**Keywords:** sensor, measurement, accelerometer, gait, biomechanics

## Abstract

There is limited validation for portable methods in evaluating high-speed running biomechanics, with inertial measurement unit (IMU) systems commonly used as wearables for this purpose. This study aimed to evaluate the validity of an IMU system in high-speed running compared to a 3D motion analysis system (MAS). One runner performed incremental treadmill running, from 12 to 18 km/h, on two separate days. Sagittal angles for the shank, knee, hip and pelvis were measured simultaneously with three IMUs and the MAS at the point of contact (POC), the timing when the foot initially hits the ground, as identified by IMU system acceleration, and compared to the POC identified via force plate. Agreement between the systems was evaluated using intra-class correlation coefficients, Pearson’s *r*, Bland–Altman limits of agreements, root mean square error and paired *t*-tests. The IMU system reliably determined POC (which subsequently was used to calculate stride time) and measured hip flexion angle and anterior pelvic tilt accurately and consistently at POC. However, it displayed inaccuracy and inconsistency in measuring knee flexion and shank angles at POC. This information provides confidence that a portable IMU system can aid in establishing baseline running biomechanics for performance optimisation, and/or inform injury prevention programs.

## 1. Introduction

Biomechanical analyses of running are frequently utilised by skilled practitioners in the sports science and medicine realm. Running forms the fundamental basis of numerous sports, and assessment may enable technical coaching to optimise performance and/or inform injury prevention programs [1,2,3]. Running analysis often relies on technology, as certain gait cycle characteristics are difficult to see with the naked eye. This becomes particularly relevant as running speed increases, when the stance phase duration of running approaches 0.1 s [4]. The ongoing challenge for practitioners and coaches is finding a field-based method of running analysis that is valid and reliable [5], noting the gold standard for high-speed running analysis is the lab-based motion analysis system (MAS). Although the MAS provides accurate measures of spatiotemporal, kinematic and kinetic parameters, its ability for use in the field or outdoors is limited. Furthermore, the MAS is expensive and time consuming as a result of extensive processing procedures [6].

Inertial measurement units (IMUs) are an emerging portable, inexpensive method for analysing running biomechanics [7]. IMUs are portable sensors made up of three components: an accelerometer, gyroscope and magnetometer which, respectively, provide three-dimensional linear acceleration, angular velocity and orientation outputs. Computational methods enable various spatiotemporal and kinetic outputs, and two or more calibrated IMUs are capable of tracking joint angles, permitting functional activity kinematic analysis [8,9].

For IMU use in running, there is not a ‘one size fits all’ algorithm; precision is determined by user requirement, and accuracy will fluctuate depending on design and calibration [10,11]. The ability of IMUs to predict running kinetics is variable [12,13], and despite emerging evidence demonstrating merit in using IMUs for kinematic analysis [14,15,16,17,18,19], the majority of studies to date have utilised IMUs for activity recognition and spatiotemporal parameters [7,20,21,22,23,24]. Furthermore, it is recognised that running biomechanics change with increasing speed [4,25,26,27]; but as velocity increases, the precision of IMU outputs may change [28,29,30]. Thus, in order to confidently utilise IMUs in the field, it is necessary to validate relevant biomechanical variables at speeds pertinent to the activity of interest.

The primary aim of this study was to validate the use of a multisensor IMU system for biomechanical lower limb kinematic analysis at point of contact (POC) during incremental high-speed running. Sagittal plane angles of the shank, knee, hip and pelvis were evaluated. The secondary aim of this study was to determine whether the existing IMU system was able to accurately measure stride time during incremental running.

## 2. Materials and Methods

The academic institution’s Human Research Ethics Board approved this protocol (project number 2018/133). One female sprinter (Age: 29 years, height: 1.65 m, weight: 58 kg) was recruited and provided informed consent to participate in two trials involving incremental running on a conventional treadmill, two weeks apart. She fulfilled the following approved inclusion criteria: aged between 18–50 years old, regularly participated (at least 1x/week) in a sport or exercise that requires high-speed running over the last 6 weeks, no musculoskeletal injury within the last 3 months, no neurological conditions, no lower extremity surgery within the last year and a body mass index less than 30. 

The IMU system trialled in this study was the Noraxon ‘MyoMOTION’ sensor and software (Noraxon USA Inc., Scottsdale, AZ, USA, Model 680 receiver, Model 610 sensor, MR 3.16 software). The IMUs used for this research had >8 h operating time (3 h to recharge), were 37.6 mm × 52 mm × 18.1 mm in size, weighed 34 g, and could sample at rates up to 200 Hz and had a Gyro speed of 2000 deg/s with an acceleration range of 16G. 

A 14 camera MAS (Eagle and Cortex 1.1.4.368, Motion Analysis Corporation, Santa Rosa, CA, USA) was utilised as the gold standard comparison for capturing 3D motion of the shank, thigh and pelvis segments at a sampling rate of 200 Hz. Positioned in the centre of the 4 m × 3 m × 3 m capture volume was a conventional treadmill (Model FQTM250, Fitquip, Scarborough, Australia). The reference gold standard comparison for POC timing was determined via a force plate (Model 9287BA, Kistler, Winterthur, Switzerland) sampling at a rate of 200 Hz mounted directly below the left rear base of the treadmill. Once the treadmill was in position, the force plate was zeroed. The IMU system and MAS force platform were time synchronised via the Noraxon MyoSync device (Noraxon USA Inc.). This device generates a pulse to synchronise signals across multiple systems.

IMUs were secured unilaterally to the runner’s left shank (Sensor 1) and left thigh (Sensor 2) with double-sided tape (Logemas Pty Ltd., Albion, Australia) and elastic adhesive bandage (Elastoplast, Beiersdorf Australia Pty Ltd., North Ryde, Australia). Sensor 1 was positioned approximately 3–4 cm above the lateral malleolus and Sensor 2 on the mid lateral thigh. The final IMU was worn centrally on the sacrum via a Noraxon myoMOTION pelvic strap (Sensor 3) and reinforced with rigid tape (Elastoplast, Beiersdorf Australia Pty Ltd., North Ryde, Australia). Vertical IMU alignment when standing was checked and ensured.

Twenty-two 12 mm retroreflective markers were adhered via double-sided tape to the pelvis and left leg (posterior superior iliac spines, anterior superior iliac spines, greater trochanter, lateral femoral epicondyle, medial femoral condyle, tibial tuberosity, head of fibula, lateral malleoli and medial malleoli, head of the first and fifth metatarsals, and the distal end of the hallux), including two 4 marker clusters on their lateral shank and lateral thigh for more accurate tracking direction relative to joint centres [31]. Foot markers were also adhered via double-sided tape to standard running shoes in the corresponding position to palpated bony landmarks.

**Figure 1:**
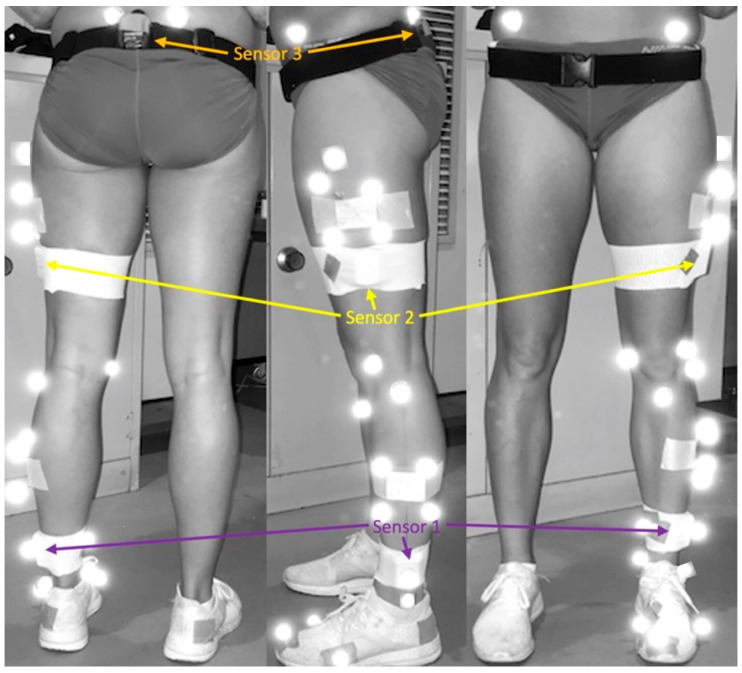
IMU and retroreflective marker set up.

**Figure 2:**
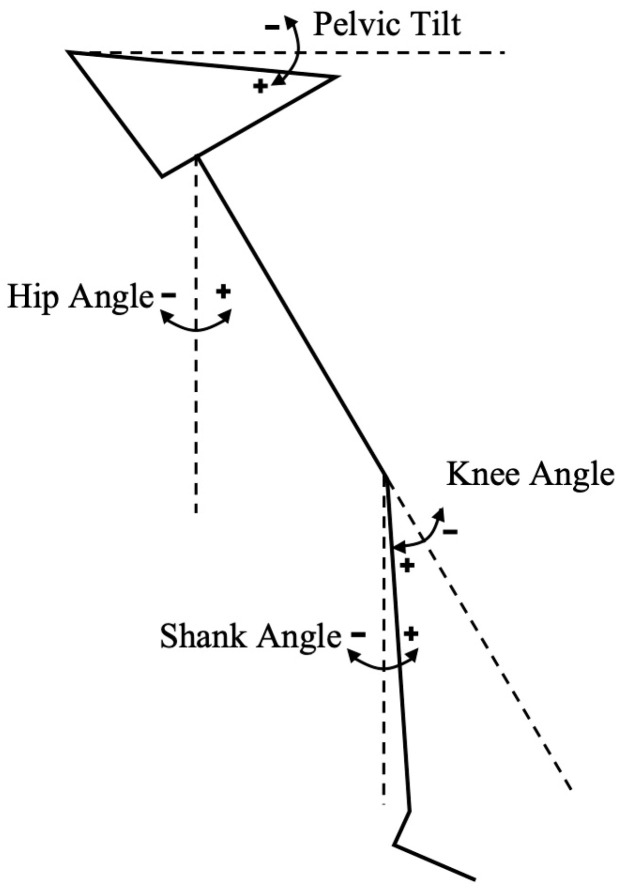
Kinematic data collected.

Once the retroreflective markers and IMUs were donned (as seen in Figure 1), calibration of both systems was conducted. Static reference data were collected simultaneously by the two systems with the runner standing on the centre of a conventional treadmill in order to determine the anatomical reference (zero) position for the lower limb kinematic data (Figure 2). A positive shank angle denoted inclination relative to the standing position. 

The runner conducted her own warm up consisting of graduated running and dynamic stretches before practicing the trial protocol. The runner was given five minutes to rest between the practice and the trial. The runner was then instructed to perform incremental running at speeds of 12, 14, 16 and 18 km/h. The runner controlled the increase in speed, and once the target speed was achieved, she was instructed to remain in a steady state (neither accelerating nor decelerating) at each speed for at least 5 s. 

Following the trial, MAS markers were reviewed and force plate POC checked for noise. Any marker switches were rectified and unidentified markers were named. The MAS trial capture of approximately 40 s (6–10 stride cycles per speed) was next exported into Visual 3D (Version 4.95, C-Motion Inc., Germantown, MD, USA) for segment definition and joint angle computation. A biomechanical model was created to develop virtual markers (based on bony landmark markers) for the pelvis, left hip joint centre, left knee joint centre, left tibial tuberosity centre and left malleolar centre. Thigh and shank clusters were utilised as target tracking markers. X, Y and Z axes in Visual 3D correspond to the medio-lateral, antero-posterior and axial anatomical axes. This biomechanical model enabled sagittal plane kinematics for the pelvis, hip, knee (Carden sequence X, Y, Z) and shank to be extracted (Carden sequence Y, Z, X). For joint calculation of the IMU data, the same segments were used.

**Figure 3:**
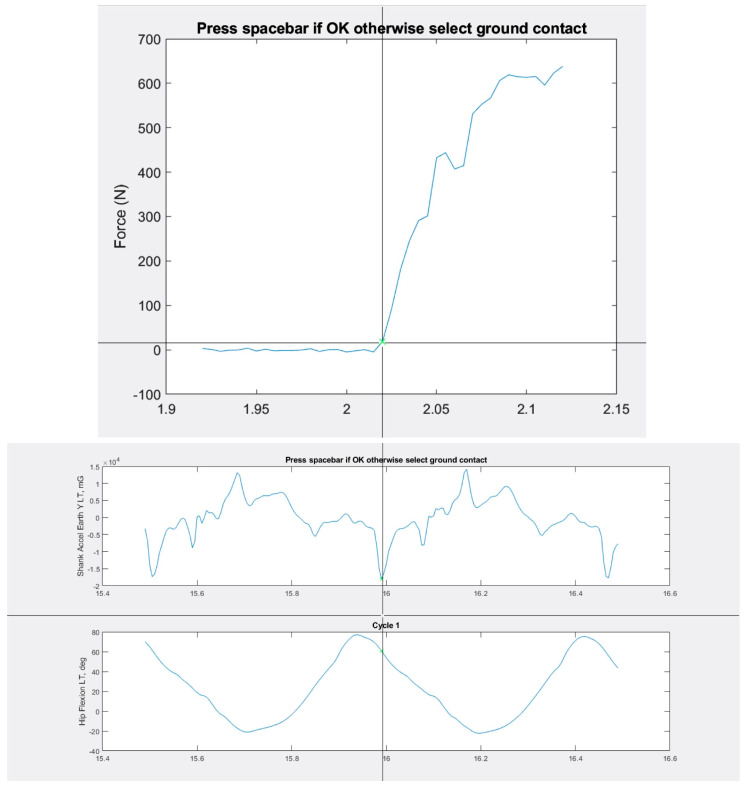
Matlab screenshots displaying Motion Analysis System point of contact identification (**top**) and Inertial Measurement System POC detection method (**bottom**).

Matlab (9.13.0 (R2022B), The MathWorks Inc., Natick, MA, USA) software was utilised for POC determination for MAS and IMU system trial data. For the MAS force plate data, POC was determined by a threshold selection of the summed vertical ground reaction force (Figure 3) and the first data point above the baseline force level. For the IMU system, POC was identified using the shank IMU’s vertical, earth-based accelerometer data (axial axes). Shank accelerometer data were chosen to minimise impact transmission time lag from the collision point/feet to pelvis [32]. The first negative minima in acceleration trace after maximal hip flexion was manually identified for each stride cycle (Figure 3). 

Biomechanical data from both systems was then exported into Excel (Microsoft Corporation, Redmond, WA, USA, 2019) for visualisation and calculation of stride time (time between respective POCs). Review of a trial video on the IMU system enabled time stamp identification of each speed. Running data during transition between speeds were removed before statistical analysis. This entire trial protocol was repeated with the same participant on a second day, 2 weeks later. Noting one stride cycle was considered one sample, and at least six strides were collected for each of the four speeds over two occasions, the sample size was deemed appropriate for at least 80% power [33,34].

Statistical analysis was conducted via SPSS Statistics (IBM SPSS Software, version 15.0). Agreement in kinematics (sagittal plane pelvic, hip, knee and shank angle), stride time and POC between the systems were evaluated using single measures 2-way mixed effects, intra-class coefficients (ICCs) for absolute agreement, Pearson’s *r* correlation (with 2-tailed significance value), root mean square error (RMSE) and paired *t*-tests. Bland–Altman Plots were used to evaluate potential directional bias for kinematic variables. 

ICC values were a priori categorised as excellent (0.90 to 1.00), good (0.75 to 0.89), moderate (0.50 to 0.75) or poor (0.00 to 0.49) [35]. The magnitude of Pearson’s *r* correlation coefficients were classified as very high (0.90 to 1.00), high (0.70 to 0.89), moderate (0.50 to 0.69), low (0.30 to 0.49) or negligible (0.00 to 0.30) [36]. We determined that a kinematic error (RMSE) between 2 and 5 degrees was considered an acceptable level based on common clinical situations [37]. We also calculated IMU system RMSE as a percentage of MAS mean in order to appreciate the clinical relevance for joint angles of varying size.

## 3. Results

**Figure 4:**
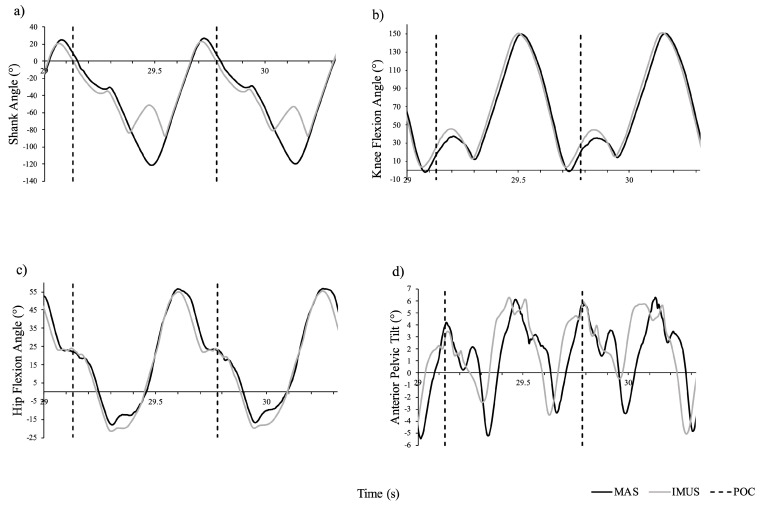
Sample sagittal angles for (**a**) shank, (**b**) knee, (**c**) hip and (**d**) pelvis as measured by the MAS (black) and the IMU system (grey) while running at 16 km/h.

**Figure 5:**
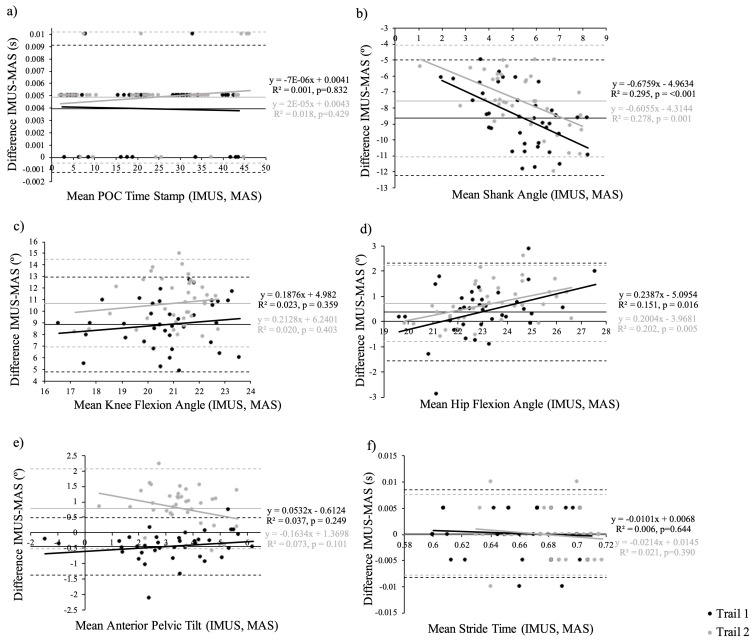
Bland–Altman Plots (mean difference plotted against the average of each measurement pair) for (**a**) point of contact (POC), (**b**) shank angle, (**c**) knee flexion angle, (**d**) hip flexion angle, (**e**) anterior pelvic tilt and (**f**) stride time for Trial 1 (black) and Trial 2 (grey).

Over 5 s at each speed (12, 14, 16 and 18 km/h), a total of 38 strides per trial, were collected. An example of kinematic data obtained through the entire stride cycle as identified by respective MAS and IMU system methods (before extraction of POC kinematic data) is displayed in Figure 4. Descriptive statistics for POC identification; sagittal plane pelvis, hip, knee and shank angle; and stride time are shown in Table 1 and Bland–Altman plots (Figure 5). Additional data provided in Appendix A include individual plots for each variable across speed.

POC was consistently determined by the IMU system (ICC 1.0 for all speeds across both trials) and had a perfect linear correlation with the MAS (Pearson’s *r* values of 1.0 for all speeds across both trials). RMSE revealed a high level of accuracy (0.0038–0.0155 s across both trials); however, the paired *t*-test revealed a significant difference for both trials (*p* < 0.030 for all speeds). This may be explained within the Bland–Altman plot, which displayed a minor offset error (0–0.01 s).

The IMU system was inconsistent in measuring shank inclination (ICC ‘poor’ at all speeds over both trials). Pearson’s *r* values were variable and tended to decrease to ‘negligible’ as speed increased to 18 km/h. Accuracy of shank inclination was also poor, with RMSE reported as 80–94% of MAS mean and significant differences in paired *t*-tests over all speeds in both trials. Both systematic and offset errors were evident on the Bland–Altman plot.

The IMU system also performed inconsistently in measuring knee flexion (ICC ‘poor’ across all speeds in both trials). Pearson’s *r* values were inconsistent and varied from ‘poor’ to ‘high’ across speeds and trials. Accuracy was likewise poor and decreased with speed (RMSE ranged from 7 to 12° or 43–82% MAS mean). The paired *t*-test also revealed a significant difference between variables over all speeds in both trials. The Bland–Altman plot displayed offset error but no consistent systematic error. 

Hip flexion was measured more consistently by the IMU system (ICC ‘high’ to ‘moderate’ with increasing speed for both trials). A strong linear correlation with the reference MAS was evident (Pearson’s *r* values were ‘very high’ or ‘high’ across speeds for both trials). The majority of paired *t*-tests were significant; however, accuracy was very good, with an RMSE of <1.4° (<6% of MAS mean) across all speeds in both trials. The Bland–Altman plot revealed minor offset error.

Sagittal plane pelvic tilt was also measured with reasonable consistency by the IMU system (ICC T1 speed 12 and 14 km/h ‘very high’, speed 16 km/h ‘high’, speed 18 km/h ‘moderate; T2 speed 12 km/h ‘poor’, speed 14 km/h ‘moderate, speed 16 and 18 km/h ‘high’). Pearson’s *r* analysis demonstrated a ‘very high’ linear correlation between the systems across both trials, with the exception of T1 speed 18 km/h and T2 speed 12 km/h, which were reported as ‘high’. Accuracy was fair, with an RMSE of <0.9° in T1 (13–22% of MAS mean) and <1.3° in T2 (9–40% of MAS mean). Paired *t*-tests revealed a significant difference across all speeds except speed 18 km/h for both trials. Minimal offset error was evident on the Bland–Altman plot.

The IMU system measured stride time with reasonable consistency as well (ICC varied between ‘moderate’ to ‘very high’ except for being ‘poor’ for Trail 2 at 18 km/h). A linear correlation was evident in all speeds across both trials (Pearson’s *r* values of ‘very high’ or ‘high’) except T2 speed 18 (Pearson’s *r* ‘negligible’). Accuracy was excellent (RMSE 0.00–0.01 s across all trials, <1% of MAS mean).

## 4. Discussion

This study assessed the validity in the use of a multisensor IMU system for biomechanical lower limb kinematic analysis at POC and stride time during incremental high-speed running. The IMU system accurately and consistently determined POC, which subsequently was used to calculate stride time. The IMU system demonstrated an acceptable degree of error and was consistent in measuring hip flexion angle and anterior pelvic tilt, but was inaccurate and inconsistent for knee flexion and shank angle.

Although our 200 Hz IMU system detected POC with excellent consistency and a very high level of accuracy, a negligible offset error of approximately one sample (0.005 s) was evident. Sinclair et al. described similar findings when validating 1000 Hz shank-mounted accelerometers for POC detection during conventional treadmill running at approximately 14 km/h. They reported very good correspondence between the reference standard force place and accelerometer methods, but noted an equivalent level of absolute error [38]. Other studies may have attempted to correct this time lag by using sensors closer to the collision point, i.e., the foot [32], though accuracy of spatiotemporal parameters with foot mounted sensors is variable and dependant on sensor position on the foot [39,40]. For this protocol, it would not be worthwhile including an additional foot sensor as, statistically, the shank sensor detected POC with a high level of accuracy. Nevertheless, algorithmic adjustment may be an option should a consistent offset (subject, speed or system specific) be considered pertinent in future trials, particularly if the IMU system has a lower sampling rate. Sampling rates of 200 Hz or greater are associated with improved agreement levels when compared to reference systems [41].

From a kinematic perspective, the ability to definitively compare our findings with other studies is challenging. A limited number of comparable studies exist within the literature [42], utilising varying IMU systems with differing technical specifications and methodologies. Nuesch et al. compared outputs from a 400 Hz IMU system against an optoelectronic MAS during conventional running. Running kinematic data were collected at a participant-selected average speed of 10.5 km/h [43]. They reported that their IMU system underestimated both hip and knee flexion angle at POC, with RMSEs of 19.3 and 36.1 degrees, respectively. Conversely, the IMU system in the current study overestimated knee flexion and demonstrated only a trivial overestimation of hip flexion at POC, but with RMSEs at 12 km/h of only 9.03 and 0.52 degrees, respectfully. Lin et al. reported that their IMU system was effective at estimating hip and knee flexion angles throughout the gait cycle on a nonmotorised treadmill, but reported poor accuracy for anterior pelvic tilt (when compared to an optical MAS). However, their participants were running faster (approximately 19 to 25 km/h), while their IMU system collected data at 100 Hz [44]. Similarly, the 500 Hz IMU system evaluated against the optoelectronic MAS in Ruiz-Malagon et al.’s validation study tested poorly for pelvic kinematic measurements during treadmill running at up to 15 km/h [45].

Our results indicate that the IMU system in this study is not recommended for sagittal plane knee and shank measurements at POC. Noting the shank IMU is a critical determinant of knee position, it is no surprise that, following poor results for shank angle, knee flexion also tested poorly. The IMU system significantly overestimated knee flexion and shank angle at POC by >7°. Particularly at the shank, this offset worsened with increased speed. Higher levels of validity seem to be associated with how close the sensor was to the area of interest [41]. This may be an explanation as to why the pelvic sensor alone accurately measured anterior pelvic tilt; the pelvic and thigh sensors accurately measured hip flexion, but the thigh and shank sensors inaccurately measured knee and shank angle. It is plausible that the shank sensor was positioned too distal on the lower leg. More research is needed to understand the effect of placement and its impact on data collection.

An interesting point to note on the example kinematic data obtained through the entire stride cycle (Figure 4) is a flip in the shank pitch angle between POCs. Although this issue was not pertinent for this study (as we were only interested in POC data), upon discussing this with the manufacturer (Noraxon USA Inc.), it appears that the matter could be related to sensor saturation and quaternions. Noraxon has stated that their newer model ‘Ultium’ has been configured to address this issue.

When interpreting the results of the present study, we must acknowledge the methodological limitations. Firstly, the single case design may affect the generalisability of findings. The results may vary with gender, age, and extrinsic (footwear, running surface) and intrinsic differences (e.g., foot strike pattern, velocity) [46]. Peak impact force will vary depending on foot strike pattern type (e.g., rearfoot, midfoot or forefoot). At slower speeds, distance runners tend to rear foot strike, whereas sprinters adopt a forefoot strike [47]. At speeds greater than 18 km/h, most runners who rearfoot strike at slower speeds transition to a mid or forefoot strike pattern [48]. Strike type tends to also be associated with footwear. Habitually shod runners mostly have a rearfoot strike, whereas runners who are habitually barefoot or wear minimalistic shoes mostly strike at the fore or midfoot [49]. 

Running surface is another factor to consider when interpreting the findings. This study was conducted on a conventional treadmill. When compared to overground, runners on conventional treadmills elicit biomechanical differences including extended step (contact) times and increased knee extension at POC. These findings can be attributed to the hypothesis that a conventional treadmill contributes to hip extension, as the belt moves the lower extremity backwards [50]. Although overground running would have been preferred, the conventional treadmill enabled greater control of running velocity in order to differentiate between speeds for the purposes of incremental validation. Additionally, environmental constraints of the current MAS laboratory disallowed the collection of multiple strides at different overground speeds.

Another important point to note is the use of externally secured markers and wearable sensors and the resultant soft tissue artifact risk. Soft tissue artifact refers to the mobility or ‘wobbling’ of markers on the skin with movement [51,52]. IMUs and clusters secured in the middle of body segments are particularly susceptible to this due to changes in muscle contour. Despite optimisation of MAS single marker position on bony landmarks, these too are subject to motion artifact with impact [53]. Thus, with increasing running speed and subsequent impacts, both systems are suspectable to measurement errors associated with soft tissue impact [54]. This issue is obviously not unique to this study alone and is a limitation that should be considered in all studies utilising wearable markers and technology.

Overall, the results of this single female participant study suggest that the IMU system described (Noraxon ‘MyoMOTION’) demonstrated appropriate agreement with the gold standard MAS for sagittal plane pelvic and hip kinematics at POC and stride time determination during high-speed running. At the same time, this study found that the IMU system described is not recommended for knee flexion or shank angle at POC. Further testing of other IMU systems and additional subjects is required to enable generalisation of findings. Nonetheless, these preliminary findings suggest that that the IMU system described enables sophisticated analysis of the validated running variables of interest out-field, which in turn can be used to optimise performance and/or inform injury prevention programs. 

## Figures and Tables

**Table 1:** ICC, Pearson correlation coefficient, RMSE and *t*-test results for the IMUS and MAS at POC.

	**Trial 1**				**Trial 2**			
	ICC (95% CI), *p* Value	Pearson’s *r*, *p* Value	IMU System RMSE (% MAS Mean)	Paired *t*-Test MAS $\bar{x}$ (SD), IMU System $\bar{x}$ (SD), *p* Value	ICC (95% CI), *p* Value	Pearson’s *r*, *p* Value	IMU System RMSE (% MAS Mean)	Paired *t*-Test MAS $\bar{x}$ (SD), IMU System $\bar{x}$ (SD), *p* Value
**POC Detection**								
12 km/h	1.00 (1.00,1.00), <0.001 *	1.00, <0.001 *	0.005 s ^	5.44 (2.12), 5.44 (2.13), 0.003 *	1.00 (1.00,1.00), <0.001 *	1.00, <0.001 *	0.0052 s ^	6.15 (2.75), 6.15 (2.75), <0.001 *
14 km/h	1.00 (1.00,1.00), <0.001 *	1.00, <0.001 *	0.0043 s ^	18.98 (2.23), 18.98 (2.24), <0.001 *	1.00 (1.00,1.00), <0.001 *	1.00, <0.001 *	0.0052 s ^	22.33 (2.29), 22.33 (2.29), <0.001 *
16 km/h	1.00 (1.00,1.00), <0.001 *	1.00, <0.001 *	0.0155 s ^	31.40 (1.97), 31.40 (1.97), <0.001 *	1.00 (0.99,1.00), <0.001 *	1.00, <0.001 *	0.0047 s ^	34.94 (1.63), 34.94 (1.63), <0.001 *
18 km/h	1.00 (1.00,1.00), <0.001 *	1.00, <0.001 *	0.0038 s ^	41.94 (1.32), 41.94 (1.32), 0.030 *	1.00 (0.99,1.00), <0.001 *	1.00, <0.001 *	0.0076 s ^	44.70 (1.19), 44.70 (1.19), 0.010 *
**POC Shank Angle**								
12 km/h	0.04 (−0.01,0.26), 0.024 *	0.61, 0.060	6.84° (94.34%)	7.25 (1.44), 0.51 (1.28), <0.001*	0.11 (−0.01,0.44), <0.001 *	0.89, <0.001 *	5.81° (76.56%)	7.59 (1.57), 1.81 (1.46), <0.001 *
14 km/h	0.03 (−0.01,0.18), 0.037 *	0.55, 0.08	8.20° (88.28%)	9.29 (1.55), 1.19 (1.21), <0.001*	0.02 (−0.01,0.14), 0.012 *	0.71, 0.015 *	7.83° (91.96%)	8.51 (1.27), 0.73 (0.81), <0.001 *
16 km/h	0.01 (−0.00,0.06), 0.680	0.48, 0.155	10.09° (88.44%)	11.41 (1.03), 1.36 (0.87), <0.001*	0.05 (−0.01,0.30), 0.004 *	0.82, 0.012 *	8.50° (80.97%)	10.50 (1.64), 2.05 (1.38), <0.001 *
18 km/h	0.01 (−0.01,0.12), 0.202	0.42, 0.35	10.13° (88.71%)	11.42 (0.89), 1.39 (1.70), <0.001*	−0.00 (−0.01,0.07), 0.580	−0.09, 0.858	10.08° (83.36%)	12.09 (1.12), 2.12 (1.22), <0.001 *
**POC Knee Angle**								
12 km/h	0.08 (−0.03,0.40), 0.030 *	0.60, 0.067	7.30° (43.17%)	16.90 (2.27), 23.98 (1.78), <0.001 *	0.06 (−0.01,0.31), <0.001 *	0.81, <0.001 *	9.03° (56.98%)	15.85 (1.96), 24.81 (1.73), <0.001 *
14 km/h	0.08 (−0.01,0.39), <0.001 *	0.81, 0.003 *	8.20° (50.37%)	16.28 (2.10), 24.39 (1.80), <0.001 *	0.00 (−0.00,0.04), 0.204	0.27, 0.429	10.89° (68.21%)	15.97 (1.10), 26.80 (0.94), <0.001 *
16 km/h	0.01 (−0.01,0.06), 0.158	0.35, 0.324	10.83° (68.59%)	15.79 (0.99), 26.55 (1.32), <0.001 *	0.00 (−0.00,0.04), 0.338	0.17, 0.68	12.11° (79.94%)	15.15 (1.29), 27.18 (0.89), <0.001 *
18 km/h	0.07 (−0.01,0.42), 0.014 *	0.81, 0.029 *	10.07° (61.74%)	16.31 (1.88), 26.26 (2.65), <0.001 *	−0.01 (−0.01,0.04), 0.915	−0.61, 0.195	12.66° (82.23%)	15.40 (1.30), 27.92 (0.94), <0.001 *
**POC Hip Angle**								
12 km/h	0.87 (0.36,0.97), <0.001 *	0.92, <0.001 *	0.86° (3.97%)	21.61 (1.63), 22.19 (1.62), 0.021 *	0.88 (0.64,0.96), <0.001 *	0.88, <0.001 *	0.52° (2.38%)	21.61 (0.94), 21.69 (1.12), 0.579
14 km/h	0.86 (0.49,0.96), <0.001 *	0.90, <0.001 *	0.68° (2.93%)	23.17 (1.26), 23.58 (1.29), 0.041 *	0.53 (−0.09,0.87), <0.001 *	0.84, <0.001 *	1.20° (5.22%)	22.95 (0.94), 24.02 (1.02), <0.001 *
16 km/h	0.69 (0.04,0.92), 0.002 *	0.84, 0.003 *	1.34° (5.66%)	23.66 (1.32), 24.59 (1.81), 0.017 *	0.70 (−0.09,0.94), <0.001 *	0.88, 0.004 *	1.39° (5.82%)	23.95 (1.60), 25.12 (1.65), 0.005 *
18 km/h	0.54 (−0.13,0.90), 0.065	0.74, 0.055	1.31° (5.80%)	22.61 (0.82), 21.93 (1.69), 0.186	0.65 (−0.09,0.94), 0.020 *	0.78, 0.066	1.02° (4.14%)	24.72 (1.17), 25.49 (1.06), 0.052
**POC Pelvis Angle**								
12 km/h	0.96 (0.43,0.99), <0.001 *	0.99, <0.001 *	0.47° (17.11%)	2.77 (1.74), 2.38 (1.77), 0.002 *	0.42 (−0.05,0.81), <0.001 *	0.88, <0.001 *	1.24° (43.04%)	2.89 (0.88), 4.06 (0.72), <0.001 *
14 km/h	0.95 (0.48,0.99), <0.001 *	0.98, <0.001 *	0.46° (17.55%)	2.61 (1.43), 2.26 (1.52), 0.003 *	0.68 (−0.04,0.93), <0.001 *	0.96, <0.001 *	1.04° (39.92%)	2.61 (1.08), 3.60 (1.11), <0.001 *
16 km/h	0.82 (0.13,0.96), <0.001 *	0.91, 0.011 *	0.86° (22.84%)	3.77 (1.30), 3.12 (1.46), 0.008 *	0.84 (0.07,0.97), <0.001 *	0.92, 0.001 *	0.89° (25.99%)	3.44 (1.46), 4.14 (1.54), 0.012 *
18 km/h	0.72 (0.11,0.94), 0.018 *	0.82, 0.023 *	0.73° (13.62%)	5.33 (0.71), 4.97 (1.14), 0.216	0.88 (0.17,0.98), 0.001 *	0.93, 0.006	0.43° (9.58%)	4.51 (0.85), 4.18 (0.85), 0.048 *
**Stride Time**								
12 km/h	0.78 (0.32,0.94), 0.003 *	0.76, 0.01 *	0.0047 s (0.68%)	0.70 (0.01), 0.07 (0.01), 0.758	0.94 (0.80,0.98), <0.001 *	0.94, <0.001 *	0.0039 s (0.56%)	0.71 (0.01), 0.71 (0.01), 1.0
14 km/h	0.88 (0.60,0.96), <0.001 *	0.87, <0.001 *	0.0040 s (0.59%)	0.67 (0.01), 0.67 (0.01), 0.724	0.87 (0.59,0.96), <0.001 *	0.88, <0.001 *	0.0034 s (0.49%)	0.69 (0.01), 0.69 (0.01), 0.676
16 km/h	0.72 (0.19,0.92), 0.008 *	0.76, 0.011 *	0.0042 s (0.64%)	0.65 (0.01), 0.65 (0.00), 0.726	0.99 (0.96,0.99), <0.001 *	0.99, <0.001 *	0.0018 s (0.26%)	0.67 (0.01), 0.67 (0.01), 0.351
18 km/h	0.89 (0.46, 0.98), 0.002 *	0.90, 0.006 *	0.0038 s (0.62%)	0.61 (0.01), 0.61 (0.01), 1.00	0.21 (−0.88,0.85), 0.346	0.19, 0.725	0.0061 s (0.96%)	0.64 (0.01), 0.64 (0.00), 0.771

^ No %MAS mean recorded as data reflects a time stamp; * *p* value < 0.05; ICC—Intraclass Correlation Coefficient; RMSE—Root Mean Square Error; $\bar{x}$—Mean; SD—Standard Deviation.

**Figure A1:**
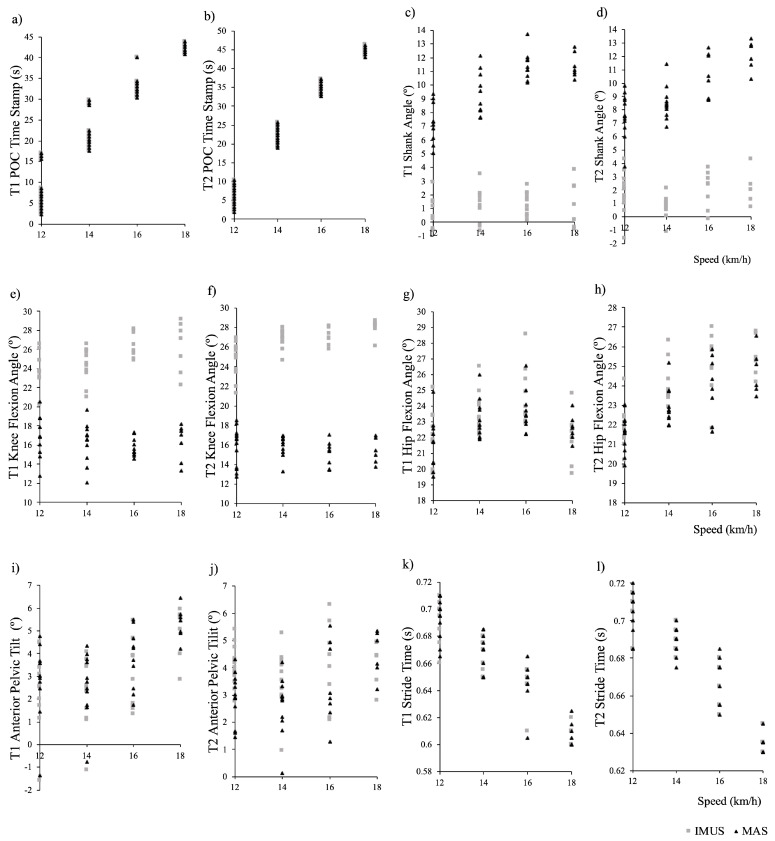
(**a**–**l**): Plots of individual study variables across speed for each trial (T1, T2). Point of contact (POC) is denoted in figures (**a**,**b**), shank angle (°) in (**c**,**d**), knee flexion angle (°) in (**e**,**f**), hip flexion angle (°) in (**g**,**h**), anterior pelvic tilt in Figures (**i**,**j**) and, stride time (s) in (**k**,**l**).

## Data Availability

The raw data supporting the conclusions of this article will be made available by the authors on request.
